# Lipoarabinomannan in Active and Passive Protection Against Tuberculosis

**DOI:** 10.3389/fimmu.2019.01968

**Published:** 2019-09-11

**Authors:** Margarida Correia-Neves, Christopher Sundling, Andrea Cooper, Gunilla Källenius

**Affiliations:** ^1^Life and Health Sciences Research Institute (ICVS), School of Medicine, University of Minho, Braga, Portugal; ^2^ICVS/3B's, PT Government Associate Laboratory, Braga, Guimarães, Portugal; ^3^Division of Infectious Diseases, Department of Medicine Solna, Karolinska Institutet, Stockholm, Sweden; ^4^Department of Infectious Diseases, Karolinska University Hospital, Stockholm, Sweden; ^5^Leicester Tuberculosis Research Group (LTBRG), Department of Respiratory Sciences, University of Leicester, Leicester, United Kingdom

**Keywords:** lipoarabinomannan, tuberculosis, Mycobacterium, glycolipid, antibodies, monoclonal, vaccine, immune response

## Abstract

Glycolipids of the cell wall of *Mycobacterium tuberculosis* (Mtb) are important immunomodulators in tuberculosis. In particular, lipoarabinomannan (LAM) has a profound effect on the innate immune response. LAM and its structural variants can be recognized by and activate human CD1b-restricted T cells, and emerging evidence indicates that B cells and antibodies against LAM can modulate the immune response to Mtb. Anti-LAM antibodies are induced during Mtb infection and after bacille Calmette–Guerin (BCG) vaccination, and monoclonal antibodies against LAM have been shown to confer protection by passive administration in mice and guinea pigs. In this review, we describe the immune response against LAM and the potential use of the mannose-capped arabinan moiety of LAM in the construction of vaccine candidates against tuberculosis.

## Introduction

Tuberculosis (TB) causes significant mortality globally, accounting for 1.7 million deaths in 2016 (1). Morbidity is also significant, with 10.4 million people estimated to be affected by the disease in 2016, and the threat to global health is further amplified by the emergence of about 600,000 cases of drug-resistant TB per year (1). One-quarter of the world's population is estimated to be infected with the causative agent, *Mycobacterium tuberculosis* (Mtb), but the majority of those infected with the bacterium do not progress to active TB. Defining the essential components of the host response that stops disease development in the majority of infected individuals is critical to the ability to stop progression in those likely to develop disease. While clear progress has been made in defining the main elements that limit bacterial growth in infected individuals, we do not have a clear model of the immune mechanisms that determine infection control vs. disease progression (2, 3). Herein we discuss a less examined aspect of the immune response to Mtb infection—the role of the cell wall glycolipids of the bacterium in the generation and regulation of the immune response.

## Mycobacterial Glycolipids

There is currently no established immunological signal that differentiates those latently infected with Mtb from those developing active disease or indeed a signal that indicates whether a vaccinated individual will be protected from infection or disease. Despite this lack of signal, it is known that the absence of a T cell response and the inability to activate macrophages are associated with the development of active disease (3). Understandably, therefore, the focus of vaccine development has been on induction of antigen-specific T cells with the ability to produce macrophage-activating cytokines. While the vaccine trials associated with this approach have yielded significant understanding of immune responses, the trials themselves have not resulted in substantially improved protection. This inability to dramatically improve upon the current vaccine approach has led to other avenues, including investigation of the ability of carbohydrate antigens to drive humoral responses. Cell wall glycolipids from Mtb such as lipoarabinomannan (LAM), its precursors the phosphatidyl-inositol mannosides (PIMs) and lipomannan (LM), as well as phenolic glycolipids (4) trehalose dimycolate (cord factor) (5) and the sulfoglycolipids (6) have all been shown to have immunomodulatory activity, with both distinct activating and repressing activity.

**Figure 1:**
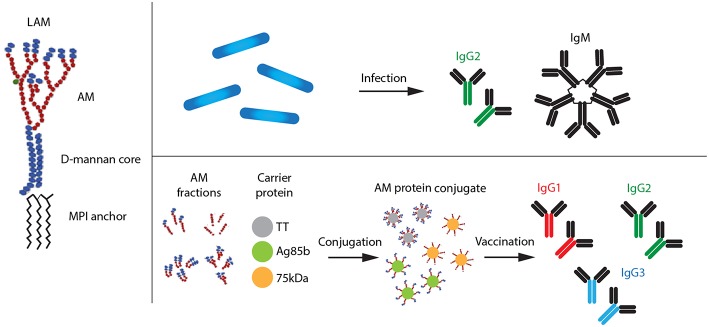
Types of LAM antibodies produced after natural Mtb infection and vaccination with AM–protein conjugate vaccine, respectively. AM, arabinomannan; MPI, mannosyl phosphatidyl-inositol.

LAM has been studied quite extensively for its immunomodulatory properties, and as a structurally unique glycolipid component of the envelope of all mycobacterial species, it provides an excellent model for discussing the role of glycolipids in immunity to Mtb (Figure 1). It is the main carbohydrate antigen of Mtb and accounts for up to 15% of the bacterial weight (7). LAM has a mannan core with a branched arabinan polymer and, in some species, capping motifs at the termini of the branched arabinan chains. The arabinan termini in Mtb are modified with caps consisting of oligomannosides, resulting in molecules designated ManLAM. LAM's mannan core is covalently linked to a mannosyl phosphatidyl-inositol (MPI) lipid moiety containing palmitic, tuberculostearic, and stearic acid residues (8–10). In addition to LAM, its precursors LM and the phosphatidyl-myo-inositol mannosides (PIMs) have been shown to have potent immunomodulatory effects on cells of the immune system (11). An extensively mannose-capped arabinomannan (ManAM) structurally related to the ManAM part of LAM is present in the capsule surrounding Mtb (12, 13).

## Innate Immune Responses to LAM

LAM has a profound effect on the innate immune response, presenting varying degrees of activity in terms of cytokine production by human macrophages and dendritic cells (11, 14, 15), but little is known about which receptors mediate this signaling. Neither is it known in most cases which parts/epitopes of the LAM molecule are important for this activity. The mannose cap of mannosylated LAM (ManLAM) was long thought to be important in the pathogenesis of Mtb infection. However, it was shown that mannose caps do not affect Mtb virulence and have minimal impact on the *in vitro* inflammatory responses in macrophages in mice (16).

A large number of host cell receptors take part in the initial interaction between mycobacteria and cells of the innate immune system (17, 18). Several classes of pattern recognition receptors are involved in this interaction, including Toll-like receptors (TLRs) and C-type lectins. Most studies report little or no TLR2 or TLR4 agonist activity of ManLAM (14, 19–22). However, ManLAM has been shown to interact with several C-type lectin ligands on cells of the innate immune system (15, 23).

ManLAM is recognized by C-type lectins that bind to carbohydrate moieties, through carbohydrate recognition domains (CRDs) (24) on the surfaces of innate immune cells (macrophages, dendritic cells, and neutrophils) (25, 26). The responses mediated by these glycan-binding receptors can take several forms. CRDs target microorganisms for uptake by endocytosis and subsequent destruction (27). Endocytic receptors, including the mannose receptor, DC-SIGN, DC-SIGNR, and langerin, interact mainly with the mannose caps on LAM. Some of these receptors also interact with PIMs and mannose-containing phenolic glycolipids. Both the mannose receptor and DC-SIGN bind tightly to LAM found on the Mtb surface, and this binding can result in efficient internalization of Mtb (28, 29).

It has become clear that some glycan-binding receptors initiate signaling responses, often resulting in the activation of several antimicrobial mechanisms by macrophages and dendritic cells, including the secretion of pro-inflammatory cytokines (24, 30, 31). ManLAM was recently identified as a ligand of Dectin-2 (dendritic-associated C-type lectin-2) (19, 24, 32). Dectin-2 is a C-type lectin that is expressed on several cell types, including dendritic cells and Mφ (19). Purified Mtb ManLAM induces signaling via Dectin-2, an activity that requires (α1 → 2)-linked dimannoside forming caps (33).

## LAM and T Cell–Mediated Immunity

Traditionally, protective immunity to TB has primarily been ascribed to T cell–mediated immune responses, with CD4^+^ T cells playing a crucial role. As Mtb is an intracellular pathogen, cell-mediated immunity characterized by interferon gamma IFN-γ-producing CD4^+^ T helper type 1 (Th1) cells is regarded as essential for TB immunity (3). The role of vaccine-induced polyfunctional T cells, co-producing several pro-inflammatory cytokines such as IFN-γ, tumor necrosis factor (TNF), and interleukin-2 (IL-2), has also been extensively evaluated as a possible correlate of protection in both animal models and humans but has not been shown to be necessary to mediate protection (34).

CD8^+^ T cells also produce IFN-γ and TNF upon Mtb infection (35–37). Moreover, CD8^+^ T cells have cytotoxic activity and are thus able to eliminate Mtb-infected cells, using mechanisms such as the Fas- and TNF-mediated killing and granule exocytosis involving release of perforin and granzyme (in humans and mice) and granulysin (in humans) (38, 39).

Although glycolipids were long considered to not induce T cell–mediated immunity, several glycolipids, including LAM and its structural variants, are known to be recognized by and activate human CD1b-restricted T cells (40–43). In addition, liposomal delivery of LAM triggers LAM-specific T cells (44). The phosphatidylinositol moiety plays a central role in the binding of LAM to CD1b, although the exact epitopes involved in this binding have not yet been defined (45). CD1 molecules are expressed on dendritic cells, certain B and T lymphocytes, and epithelial cells (43, 46, 47). Upon recognition of mycobacterial lipids, CD1-restricted T cells can become activated and proliferate, secreting pro-inflammatory cytokines such as IFN-γ, and express cytolytic and bactericidal activity (41–43). CD1-restricted T cells have further been shown to support plasma cell formation and survival in mouse models, indicating a direct role in B cell responses (48).

An interesting strategy to search for immunological mechanisms/markers that predict protection against TB is to study the immune response in individuals with latent TB infection (LTBI) in whom the infection is controlled (49–52). Using this strategy, a correlation between the frequency of LAM-specific cytotoxic CD8^+^ T cells and the ability to control infection was found in subjects latently infected with Mtb (52). These IFN-γ-producing CD1b-restricted CD8^+^ T cells coexpressed perforin, granulysin, and granzyme B and limited bacterial growth *in vitro*, suggesting a possible mechanism of protection against TB (52). In contrast, a study using a whole blood assay stimulated with LAM showed a reduced induction of TNF and IFN-γ and a higher production of IL-10 in TB patients and contacts compared to healthy controls (53), indicating a complicated interplay between LAM and the immune system. It is now clear that LAM and other glycolipids play an important role in the immune response to infection by Mtb. Understanding to what extent T cell responses to these molecules are of relevance for the establishment of a protective immune response needs to be definitively addressed.

## Humoral Immunity Induced by LAM and the Role of B Cells

As Mtb is a facultative intracellular pathogen, it has been assumed that antibodies (Abs) play no relevant role in the protection against TB and may even contribute to immunopathology (54). However, emerging evidence suggests that B cells and humoral immunity can modulate the immune response to various intracellular pathogens, including Mtb (55–59). Abs have been shown to confer protection against a number of intracellular pathogens by modulating immunity via Fc receptor–mediated phagocytosis, antibody-dependent cellular cytotoxicity (ADCC), and immunostimulation (57).

It has been shown that B cells can regulate the level of the granulomatous reaction, cytokine production, and the T cell response during Mtb infection (55, 56, 58–60). However, the regulation is complex due to the impact of both secreted products and direct cellular interactions. For example regulatory B cells, which produce IL-10 or transforming growth factor β, participate in the immunomodulation of immune responses, and it was recently shown that ManLAM induced IL-10 production by B cells, both *in vitro* and *in vivo*, predominantly through TLR2 (61).

It was recently shown that some B cells are functionally altered in active and latent TB (62). These “atypical” B cells displayed impaired proliferation, cytokine production, and differentiation to Ab-secreting cells. Similar atypical B cells have been described in several other infectious diseases and were shown to be rapidly induced and then accumulate, especially during chronic or recurrent infections, such as in malaria and infection by hepatitis B and C virus infection and HIV (63–65). The role of atypical B cells in immunity remains unclear. Due to a reduced capacity to respond to B cell receptor stimulation and differentiation to Ab-secreting cells *in vitro* (66–68), high levels of atypical B cells have been suggested to be detrimental for the pathogen-specific immune response (63). It remains to be determined if important protective Mtb-specific B cell reactivities are sequestered away within the atypical B cell compartment, where their contribution to the circulating Ab pool might be limited.

### Anti-LAM Antibodies

It has long been known that Abs against mycobacterial antigens are formed during natural infection (57, 69). In a recent study, IgG levels against 15 Mtb protein antigens were shown to be higher in TB patients than in endemic and non-endemic healthy IGRA-negative controls, with one exception, Abs against the latency antigen Rv1733c that were preferentially present in endemic controls (70). During active TB, disease production of Abs is prominent (71, 72), but such Abs have been reported to have low avidity and low IgG/IgM ratio (73), potentially indicating low contribution of germinal center–derived B cell responses. However, there is evidence to suggest that Abs may limit the dissemination of Mtb and potentially also play a role in prevention of infection via mucosal immunity (74). In a few animal studies, Abs against Mtb have been shown to be protective against Mtb infection (75, 76). In addition, a human IgA monoclonal Ab (mAb) to 16 kDa α-crystallin (Acr) from Mtb reduced bacterial load in mice transgenic for the human FcαR1 receptor (75). Coating bacille Calmette–Guerin (BCG) with mAbs to the heparin-binding haemagglutinin adhesin (HBHA) was also shown to impair dissemination after intranasal infection (77).

Anti-LAM Abs are induced both during Mtb infection (73, 78, 79) and after BCG vaccination (80–82). The titers of Abs against LAM and capsular arabinomannan (AM), were significantly lower in HIV-infected than in HIV-non-infected TB individuals, which might be due to impaired B cell function in HIV-positive patients (83–85). In children, an inverse correlation was detected between the presence of anti-LAM IgG Abs and disseminated TB, suggesting that LAM Abs might be important in limiting TB dissemination (86).

Anti-LAM Abs induced during infection have been associated with bacterial opsonization and restriction of intracellular growth (81, 82). Opsonization with anti-LAM Abs from latently infected individuals was shown to enhance uptake and killing of Mtb (87) and BCG (81) by donor macrophages. Key factors of macrophage microbicidal action, such as increased production of pro-inflammatory cytokines like IFN-γ and IL-6, phagosome acidification, and nitric oxide production, through expression of inducible nitric oxide synthase, were also significantly enhanced following Ab opsonization (87).

#### Anti-Mtb Ig Isotypes and Immune Response to Mtb

The activity of anti-Mtb Abs is strongly affected by their isotype (88). Distinct Ab glycosylation patterns have gained increasing attention as regulators of immune responses (49, 89). In patients with TB, the main Ig isotype response to polysaccharides (PSs), including LAM, is IgG of predominantly the IgG2 subclass (83, 85). This is in accordance with the general observation that Abs to carbohydrates tend to be IgG2 (90–92).

Vaccination of cattle with BCG induces both IgG1 and IgG2 Abs to ManLAM, which are transferred in colostrum to neonatal calves (93). LAM-specific IgA Abs can also be induced through oral vaccination with BCG (80). This is important as IgA, but not IgG, Abs specific for different Mtb surface antigens including ManLAM can block Mtb uptake by lung epithelial cells, independent of the expression of IgA Fc receptors (88).

Human levels of LAM-specific IgG2, but not IgM, correlate positively with classical complement activation induced by BCG (94). The binding of complement to BCG incubated with purified rabbit anti-LAM Abs followed by factor B–depleted serum indicates that anti-LAM IgG bound to the bacilli can mediate classical complement activation and deposition on the mycobacteria (94).

### Monoclonal Antibodies Against LAM

Monoclonal Abs (mAbs) are increasingly used for therapeutic purposes. Yet, despite the clear role of Abs in many infections, only two monoclonal therapeutic Abs against infectious agents (human respiratory syncytial virus and *Bacillus anthracis*) have been licensed (95). There are, however, a number of therapeutic Abs against infectious diseases under development, mainly against viral diseases but also some bacterial agents (both Gram positive and Gram negative) and fungi (95).

Over the years, several mAbs have been raised against ManLAM [(76, 96–98); Table 1]. Some of these were shown to confer protection by passive administration in mice [(76, 96, 97, 99); Table 1]. However, these studies provided very little information about possible mechanisms of protection. Two mAbs, SMITB14 and 9d8, were shown to prolong survival of mice. Given intravenously to mice previously infected with Mtb (strain Harlingen), mAb SMITB14 reduced bacterial load and weight loss (76). The fact that the Fab2 fragments of SMITB14 showed a similar inhibitory effect as the full Abs indicates that the specific epitope targeted mediated the effect, rather than Fc-mediated functions. Incubation of Mtb (strain Erdman) with mAb 9d8, before intratracheal challenge, was shown not to reduce the bacterial load in the spleen, liver, or lungs (96). However, it profoundly altered the nature of the granulomas in the lungs, where bacilli localized within the center of well-organized granuloma. This suggests that the mAb conferred protection by enhancing the cellular immune response (96). Together, these observations suggest that targeting ManLAM can mediate protection via different mechanisms. In support of this, a single-stranded DNA aptamer that specifically binds to ManLAM, isolated from the virulent Mtb strain H37Rv, reduced Mtb infection in mice and rhesus monkeys (100).

**Table 1:** *In vivo* effect of anti-LAM/AM monoclonal antibodies.

**Antibody**	**Isotype**	**Epitopes**	**Administration route**	**Mouse strain**	**Mtb challenge strain**	**Challenge route**	**Biological effect**	**Comments**	**References**
9d8	IgG3	AM	Together with challenge bacteria	BALB/c C57BL/c	Mtb Erdman	Respiratory	Prolonged survival	Bacteria were localized in center of granuloma.	(96)
9d8	IgG3	AM	NA	NA	NA	NA	NA	Binding of human Abs to AM inhibited by mAb 9d8 in patients with TB.	(13)
Sc11	IgM	AM, LAM, AG	NA	NA	NA	NA	LAM cleared from circulation when given i.v.	Deposition in spleen was reduced, while LAM deposition in the liver increased.	(97)
SMITB14	IgG1	AM	Intravenously	BALB/c	Mtb Harlingen	Intravenous	Increased long-term survival	Reduction in bacterial load in spleen and lung, decreased body weight.	(76)

*Abs, antibodies; AG, antigen; AM, arabinomannan; Ig, immunoglobulin; i.v., intravenously; LAM, lipoarabinomannan; mAb, monoclonal Ab; Mtb, Mycobacterium tuberculosis; NA, not applicable; TB, tuberculosis*.

While the majority of natural Abs against carbohydrate antigens are IgG2, most therapeutic Abs, licensed or in development, are of the human IgG1 subclass (95). Interestingly, none of the LAM-specific mAbs so far reported to induce protection against Mtb are of the IgG2 isotype (Table 1); one (SMITB14) was of the IgG1 type (76) and one (9d8) of the IgG3 type (96), indicating that these latter isotypes might be of importance in the design of protective Abs.

The affinity of IgG subclasses for varying FcγRs results in differing effector responses and has been made use of in the development of therapeutic Abs and vaccines (101). Signaling through FcγRs can modulate immunity upon infection with Mtb and significantly affect outcome (102). As a further complicating factor, in a murine model of progressive TB, the protective effect of the passive transfer of Abs depended on the glycosylation pattern of the Fc region of IgG Abs (103). Thus, the design of LAM-specific Abs with particular Fc characteristics should be explored for protective effect, including immunization strategies designed to augment cellular immune responses. Interestingly, it was recently reported that individuals with LTBI and active TB show different Mtb-specific humoral responses, where LTBI is associated with Ab Fc functional profiles associated with enhanced binding to FcγRIII, and distinct Ab glycosylation patterns (49). In comparison to Abs from active TB patients, Abs from LTBI drive enhanced phagolysosomal maturation, inflammasome activation, and in particular, macrophage killing of intracellular Mtb (49). These findings indicate a potential role for Fc-mediated Ab effector functions in the control of Mtb, by differential glycosylation.

### LAM Epitopes Recognized by Antibodies

The Abs against LAM/AM are heterogenous and differ in their specificity (13). Serum from mice immunized with AM conjugated to carrier proteins such as the Mtb antigen Ag85B or *Pseudomonas aeruginosa* exoprotein A (rEPA) (see ahead) recognizes a broad range of AM structural variants and specifically recognized arabinan fragments (104). The specificities of anti-LAM mAbs differ in their dependence on arabinofuranoside branching and capping motifs, and their specificities could be divided into two general groups: one dependent on the poly-Ara*f* backbone of LAM and the other dependent on specific mannose-capping motifs (98).

## The Potential Role of LAM in the Design of TB Vaccines

The live attenuated BCG is still the only vaccine available against TB, but it has little effect on preventing pulmonary TB in adults (105). Since pulmonary TB is the most significant source of TB transmission, BCG is thought to have limited effect on the spread of TB. During the last decades, great efforts have been made to design new and better TB vaccines (106, 107). The vaccine candidates are intended to either replace BCG or to act as booster vaccines after infant BCG vaccination. To date, essentially all subcellular vaccines tested have been based on dominant antigens of a protein/peptide nature, such as the Mtb protein antigen 85A (Ag85A) or ESAT-6 (108), with induction of potent Th1 cell responses used as an immunological correlate of protection.

The first vaccine candidate to be tested in clinical trials was MVA85A, a recombinant strain of Modified Vaccinia virus Ankara expressing Ag85A. Several trials reported its efficacy in terms of protection in animal models (109) and safety and immunogenicity in humans. However, in a phase 2b trial conducted in South Africa with infants previously vaccinated with BCG, the vaccine was well-tolerated and immunogenic but failed to confer better protection against TB compared to BCG alone (110). In another study in South Africa, also with infants who were vaccinated with BCG, there was no correlation between the number of BCG-elicited T cells that produced IFN-γ or multiple cytokines (such as IFN-γ, IL-2, and TNF) and the development of active TB (111). An important message from these studies is that the immune markers that had been used in most previous preclinical studies as correlates of vaccine-induced protection show no correlation with actual protection. Thus, it is unknown which immunological parameters or biomarkers predict who is protected from infection or who will control the infection or develop clinical disease, both in natural immunity and in vaccine-induced immunity (112).

### Carbohydrate Antigens as Vaccine Candidates

The possibility of using carbohydrate antigens as vaccine components in a TB vaccine is an interesting strategy (113). Some of the most successful vaccines against bacterial infections are based on carbohydrate antigens that are conjugated to a carrier protein. Glycoconjugate vaccines toward encapsulated *Haemophilus influenzae* type b (Hib), *Streptococcus pneumonia*, and *Neisseria meningitidis* have all been licensed (114). Additionally, a conjugate vaccine against *Salmonella typhi*, a facultative intracellular pathogen, is being developed and has shown >50% protection in a phase 2b challenge, leading to its licensing (115).

Pure oligosaccharides are poor immunogens, as they fail to recruit CD4^+^ T cell help. They are therefore limited to T cell–independent B cell immune responses. Children under 2 years are not capable of producing effective Abs against PS antigens (116). However, conjugating a bacterial PS to a carrier protein that provides T cell epitopes creates a T cell–dependent antigen, which can induce protective immunity in infants. Interestingly, during infections with PS-encapsulated extracellular bacteria, PS-specific IgG responses are largely CD4^+^ T cell dependent (117), indicating that in the presence of whole bacilli, T cell–dependent B cell responses are favored over T cell–independent B cell responses.

### Conjugate Vaccine Candidates Based on AM

AM isolated from LAM, from the bacterial cell wall (118, 119), or from the Mtb capsule (99, 104) conjugated to various carrier proteins has been used to create a number of conjugate vaccine candidates (Table 2). In a series of experiments, AM-derived oligosaccharides were covalently linked to Mtb proteins (Ag85B or a 75 kDa antigen) or tetanus toxoid (TT) and tested for their protective effect in mice (C57BL/6) and guinea pigs. Using a subcutaneous priming-intranasal boost regime, these conjugate vaccines showed good protective efficacy in mice after intranasal challenge with Mtb Harlingen and in guinea pigs after aerosol infection with H37Rv, with prolonged survival and reduced weight loss, similar to that observed after vaccination with BCG (118). Intranasal vaccination with AM–TT also had a slight effect in mice as a boost after BCG prime, with reduced bacterial load in the spleen and reduced granulomatous inflammation in the lungs (120). However, in a second set of experiments in guinea pigs, using the same infection model as previously, the vaccine candidate did not perform as well (121). The vaccine response only led to a modest reduction of bacterial load in spleens, and the guinea pigs showed poor survival compared to previous studies (118). It is possible that the route of immunization and choice of adjuvant could impact the type (IgG vs. IgA and cell mediated vs. Abs) and location (mucosal vs. systemic) of the immune response. In one study, guinea pigs were immunized subcutaneously on day 0 and boosted once intranasally on day 24 (118), while in the other, mice were immunized subcutaneously once and boosted twice intranasally 3 weeks apart (121). There was also a difference in the formulation of the adjuvant in terms of emulsion vs. suspension (see ahead).

**Table 2:** AM conjugate vaccines.

**Vaccine**	**Animal model**	**Immunization route**	**Adjuvant**	**Challenge route**	**Vaccine-challenge interval**	**Prolonged survival**	**Reduced cfu spleen**	**Reduced cfu liver**	**Reduced cfu lung**	**Other**	**References**
AMOs–TT	Mouse	s.c., boosted nasally after 6 wks	L3 (in emulsion or suspension)	i.n. Mtb Harlingen 10^5^	24 day	Yes (more so for L3 in emulsion)	ND	ND	ND	Reduced weight loss	(118)
AMOs–Ag85B	Mouse	s.c. × 2 (27 days apart)	Alum	i.v. H37Rv 10^5^	32 day	Yes	ND	ND	ND	Reduced weight loss	(118)
AMOs–Ag85B	Guinea pig	s.c., boosted nasally after 24 days	L3	Aerosol H37Rv 10 cfu per lung	6 weeks	No	Yes	ND	No	Reduced pathology in lung and spleen	(118)
AM–rEPA	Mouse	s.c., boosted s.c. × 2 at 2 and 4 weeks	Freund's incomplete	i.v. Mtb Erdman 10^6^	6 weeks?	No	ND	No	Yes	Early moderate reduction of cfu	(99)
AM–rEPA	Mouse	s.c., boosted s.c. × 2 at 2 and 4 weeks	Freund's incomplete	i.v. BCG 2.5 × 10^5^	6 weeks?	No	Yes (*p* = 0.025)	Yes (*p* = 0.01)	No		(99)
AM–TT	Mouse	i.n. 6 months after BCG	L3	i.v. H37Rv 3 × 10^5^	10 weeks	ND	Yes	ND	No	Reduced granulomatous inflammation in lungs	(120)
AMOs–Ag85B	Guinea pig	s.c. boosted nasally 3 wks later	L3	Aerosol H37Rv 10–50 cfu/lung	6 weeks	No	Yes	No	No		(121)
AMOs–TT	Guinea pig	s.c. and boosted × 2 nasally 3 weeks apart	L3	Aerosol H37Rv 500 cfu	8 weeks	No	No	No	No		(121)
AM–Ag85B	Mouse	i.p. × 3 (2 weeks apart)	Alum	Aerosol H37Rv 100 cfu	4 weeks	Yes, similar to BCG	Yes	ND	Yes	Reduction in diseased lung tissue	(104)
AM–PA	Mouse	i.p. × 3 (2 weeks apart)	Alum	Aerosol H37Rv 100 cfu	4 weeks	Slight increase in survival	Yes	ND	Yes	Reduction in diseased lung tissue	(104)

*Ag, antigen; AM, arabinomannan; AMOs, AM oligosaccharide; BCG, Bacillus Calmette-Guérin; cfu, colony forming units; i.n., intranasally; i.p., intraperitoneally; i.v., intravenously; LAM, lipoarabinomannan; ND, not done; rEPA, Pseudomonas aeruginosa exoprotein A; s.c., sub-cutaneously; TT, tetanus toxoid*.

Similarly, AM–rEPA conjugates were used for subcutaneous vaccination of mice, resulting in a moderate effect on the bacterial load in the lungs but not in the spleen early after challenge with Mtb Erdman (99). In a more recent publication, intraperitoneal immunization with either AM–Ag85b conjugate or AM conjugated to *Bacillus anthracis* protective antigen (PA) formulated in alum resulted in a reduced bacterial load in the lungs and spleen and modest increased survival after aerosol challenge with Mtb H37Rv (104). Thus, AM conjugated to various carrier proteins, using different vaccination protocols, showed protection against Mtb challenge. In some cases, the protection was modest, and it never surpassed the effect of vaccination with BCG.

### Immunogenicity of AM Conjugate Vaccines

The mechanisms of the (partial) protection induced by AM conjugate vaccines reported so far are not clear. Most conjugate vaccines licensed to date are thought to work by inducing protective Abs. In accordance, neutralizing Ab titers are often used as correlates of protection (122). AM oligosaccharide conjugates were highly immunogenic in rabbits (119), and various AM oligosaccharide–protein conjugates were shown to induce robust LAM-specific IgG in mice (123), all inducing a boostable T helper cell–dependent IgG response.

Ab responses were not investigated in the few early protection studies with AM conjugates. More recently however, immunization with either AM–Ag85b or AM–PA conjugates was found to elicit an AM-specific Ab response in mice that were partially protected against Mtb challenge (104),

Abs raised after vaccination with conjugate vaccines are mostly of the IgG1 subclass. For example, the dominant subclass was IgG1 in subjects vaccinated with pneumococcal TT conjugate, whereas, as expected, Abs induced by pneumococcal PS had a higher proportion of IgG2 (124). A mix of IgM, IgG1, and IgG2b was observed in the AM–Ag85b immunized mice vs. an exclusive IgG2b Ab response in AM–PA immunized mice, in both cases with alum as an adjuvant (104).

## Factors to Consider for the Design of AM Conjugate Vaccines

The immunogenicity of conjugate vaccines depends on the nature and size of the PS, the nature of the carrier protein, the conjugation method and the adjuvant (125).

### Size of Carbohydrates

AM oligosaccharides with an apparent molecular weight of 5.2 kDa determined by gel filtration coupled to carrier proteins were recognized by LAM-specific mAbs, irrespective of coupling procedure or carrier protein used (119). Conjugates with AM oligosaccharides with an apparent molecular weight of 28 kDa reacted with the mAbs. However, none of the conjugates containing smaller-sized (apparent molecular weight 1.0 kDa) AM fragments reacted with the mAbs, indicating that the epitopes were absent or destroyed during the conjugation process.

#### The Role of Carrier Protein

In the choice of carrier proteins, several considerations should be taken into account, in particular, potential pre-exposure or co-exposure to a given carrier, which may lead to immune interference and reduction of the anti-carbohydrate immune response, and the dual role of proteins as carrier and protective antigen (126). Combining AM with the mycobacterial antigen Ag85B or the 75 kDa antigen induces a higher protection than AM conjugated to a non-mycobacterial antigen, such as TT (118) or rEPA (104). This could indicate that the mycobacterial protein antigens serve not only as carrier proteins but also as efficient immunogens on their own. On the other hand, it could also indicate that the mycobacterial antigens are more efficient carrier proteins compared to TT.

#### Conjugation Method

In the construction of AM–protein conjugate vaccines, two different methods for conjugation of AM oligosaccharides to carrier proteins were evaluated: direct reductive amination (127) and a thioether linkage method (128). Using oligosaccharide fragments with an apparent size range of 1.0–5.2 kDa, both methods yielded oligosaccharide–protein conjugates with a similar degree of substitution. The thioether linkage method was also efficient for conjugation of AM oligosaccharides of higher apparent molecular mass (28 kDa) to both TT and cross-reacting material 197 (CRM197), while direct reductive amination was not effective in conjugating oligosaccharide fragments of apparent molecular mass of 28 kDa. This is most probably due to the linkage arm used in the thioether linkage method, circumventing steric hindrance during conjugation. The choice of the carbohydrate site for conjugation to carrier proteins heavily impacts the immune response. Upon conjugation to semi-synthetic LAM derivatives, starting from mannose and two disaccharide analogs, Ag85B maintains its ability to activate B cells after glycosylation while showing a significant reduction in stimulating T cell response (129).

#### The Role of Adjuvants

Adjuvant use impacts on the type and magnitude of the immune response induced. Alum compounds classically induce strong Ab responses, while emulsion-based adjuvants generate a more balanced humoral and cell-mediated immune response (130). In a study on the efficacy of various adjuvants using H56 (Ag85B–ESAT-6–Rv2660c) as an antigen, the highest level of protection was seen upon vaccination with H56 in combination with emulsion-based adjuvants generating Th1 responses, while H56 combined with alum was not protective (130). This could indicate that vaccine-induced Abs only play a minor role in the vaccine-induced protection against Mtb, but it is also possible that qualitative features induced by the different adjuvants have an important effect on the protective response. In support of this hypothesis, it was recently shown that Abs with improved binding to FcγRIII are associated with better control of Mtb infection in humans (49). The Ab isotypes primarily interacting with FcγRIII are IgG1 and IgG3, which are normally induced in response to intracellular pathogens via the secretion of Th1 cytokines. However, since LAM-specific Abs are commonly of the IgG2 subtype (83, 85), characteristic of a Th2-derived immune response, a novel vaccine would likely benefit from directing the Ab response toward a more balanced Th1/Th2 profile. Consistent with this, a TB vaccine using alum as an adjuvant did not perform as well-compared to when given with the liposomal L3 adjuvant (118). Using L3 in emulsion as an adjuvant was also shown to be more effective compared to L3 in suspension (131).

Liposomes are adjuvants based on different types of lipid formulations, which can either be given together with the antigen in solution or formulated within lipid nano-structure (132). Their mechanism of action remains unclear, although liposomes stimulate a strong pro-inflammatory immune environment at the site of injection, with efficient uptake of antigen by primarily monocytes and dendritic cells (133, 134). Although liposomal adjuvant MF59 is more efficient compared to alum at this process, the addition of a TLR ligand to alum can enhance the response to levels similar to MF59 (134).

Purified TLR ligands can be given together with vaccines or other adjuvants to modulate the immune response toward a certain response pattern or increase the immunogenicity of an antigen (135). The addition of TLR ligands to alum was shown to greatly enhance immune responses in both non-human primates and humans (136, 137). By combining liposomal adjuvants with TLR ligands, the immunostimulatory effect can be improved even further, as shown by responses to the hepatitis B surface antigen and malaria RTS,S vaccine (138, 139). This allows for reduction of each vaccine dose but also modulation of the immune response toward a more balanced immune profile, with improved functionality of both T cells and B cells, which is likely an important factor for an improved TB vaccine.

#### Route of Immunization and Immunization Regimens

Most TB vaccine candidates have been developed for parenteral use. However, several studies have used mucosal delivery (140) such as intranasal immunization (141), in particular to boost parenteral BCG immunization (142, 143). Intranasal administration of an AM–TT conjugate after BCG vaccination enhanced protection in the spleen of mice, indicating a systemic protective effect, but did not reduce the bacterial load in the lung, compared to non-boosted BCG vaccinated mice [(120); Table 2]. However, the extent of granulomatous inflammation in the lungs was significantly reduced.

The number of immunizations as well as the intervals between them may also be of importance. Various methods for slow vaccine release have been explored. Interestingly, two methods of slow delivery immunization were recently shown to enhance neutralizing HIV Abs and germinal center responses in rhesus monkeys, compared to conventional immunization strategies (144), indicating the importance of how the vaccine is delivered in the resulting immune response.

A few studies in guinea pigs have explored the potential use of mycobacterial glycolipids (145, 146) as vaccine candidates, including LAM (147). A LAM extract from *Mycobacterium paratuberculosis* was shown to induce specific Abs in cattle that, after challenge, reduce *M. paratuberculosis* excretion at least for 100 days (147). We found that native purified LAM does not induce IgG immune responses in mice that can be further boosted, indicating a lack of antigen-specific memory B or T cells. This is likely due to mice only expressing CD1d, which has not been found to present mycobacterial glycolipid antigens.

## Concluding Remarks

Our increasing understanding of the immune response to Mtb glycolipids indicates that these responses play an important role in protection against TB. LAM-specific T cells have a direct role on infected cells and support the differentiation of LAM-specific B cells into plasma cells. LAM-specific Abs were shown to mediate moderate protection against Mtb infection in mouse models and correlate with better disease control in humans. However, the mechanism of protection induced by AM–protein conjugates should be further studied for the development of novel vaccination strategies, alone or in combination with mycobacterial protein antigens. Increasing knowledge on how to modulate the immune response toward a more optimal T and B cell response is necessary for the development of such a vaccine. Special effort should address the impact of adjuvants and immunization strategies but also the precise mechanisms by which LAM-specific Abs mediate protection, exploring not just the specific epitopes but also Ab Fc characteristics.

## Author Contributions

GK and MC-N decided on the structure and general content of the manuscript. GK, MC-N, and CS collected the data, summarized the information, and wrote the different chapters. AC performed an overall revision and provided important contributions that improved the manuscript. All authors discussed and commented on the structure and the content of the manuscript.

### Conflict of Interest Statement

The authors declare that the research was conducted in the absence of any commercial or financial relationships that could be construed as a potential conflict of interest.
